# A site-differentiated [4Fe–4S] cluster controls electron transfer reactivity of *Clostridium acetobutylicum* [FeFe]-hydrogenase I†

**DOI:** 10.1039/d1sc07120c

**Published:** 2022-03-25

**Authors:** Carolyn E. Lubner, Jacob H. Artz, David W. Mulder, Aisha Oza, Rachel J. Ward, S. Garrett Williams, Anne K. Jones, John W. Peters, Ivan I. Smalyukh, Vivek S. Bharadwaj, Paul W. King

**Affiliations:** National Renewable Energy Laboratory Golden Colorado USA paul.king@nrel.gov; Department of Physics, University of Colorado Boulder Boulder Colorado USA; School of Molecular Sciences, Arizona State University Tempe Arizona USA; Sandia National Laboratories Albuquerque New Mexico USA; Institute of Biological Chemistry, Washington State University Pullman Washington USA; Renewable and Sustainable Energy Institute, National Renewable Energy Laboratory and University of Colorado Boulder Boulder Colorado USA

## Abstract

One of the many functions of reduction–oxidation (redox) cofactors is to mediate electron transfer in biological enzymes catalyzing redox-based chemical transformation reactions. There are numerous examples of enzymes that utilize redox cofactors to form electron transfer relays to connect catalytic sites to external electron donors and acceptors. The compositions of relays are diverse and tune transfer thermodynamics and kinetics towards the chemical reactivity of the enzyme. Diversity in relay design is exemplified among different members of hydrogenases, enzymes which catalyze reversible H_2_ activation, which also couple to diverse types of donor and acceptor molecules. The [FeFe]-hydrogenase I from *Clostridium acetobutylicum* (CaI) is a member of a large family of structurally related enzymes where interfacial electron transfer is mediated by a terminal, non-canonical, His-coordinated, [4Fe–4S] cluster. The function of His coordination was examined by comparing the biophysical properties and reactivity to a Cys substituted variant of CaI. This demonstrated that His coordination strongly affected the distal [4Fe–4S] cluster spin state, spin pairing, and spatial orientations of molecular orbitals, with a minor effect on reduction potential. The deviations in these properties by substituting His for Cys in CaI, correlated with pronounced changes in electron transfer and reactivity with the native electron donor–acceptor ferredoxin. The results demonstrate that differential coordination of the surface localized [4Fe–4S]His cluster in CaI is utilized to control intermolecular and intramolecular electron transfer where His coordination creates a physical and electronic environment that enables facile electron exchange between electron carrier molecules and the iron–sulfur cluster relay for coupling to reversible H_2_ activation at the catalytic site.

## Abstract 2

Histidine coordination of the distal [4Fe–4S] cluster in [FeFe]-hydrogenase was demonstrated to tune the cluster spin-states, spin-pairing and surrounding molecular orbitals to enable more facile electron transfer compared to cysteine coordination.

## Graphical abstract

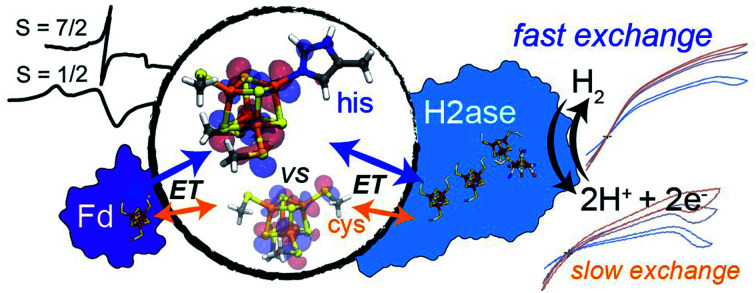

## Introduction

There is broad interest in understanding reduction–oxidation (redox) enzymes that catalyze the diverse chemical transformations that comprise biological energy transformation. Many of these reactions are relevant to energy storage in energy carrier compounds such as hydrogen gas (H_2_) or production of commodity chemicals like NH_3_ or formate. Understanding the underlying mechanistic principles of these reactions has inspired the creation of numerous synthetic catalysts within the field of biomimetic chemistry. For example, great advances in catalyst design have been achieved through a rigorous biophysical and structural investigation of the active site of hydrogenase enzymes.^1–4^ Inherent to H_2_ formation as well as all redox catalytic reactions is the process of directing electrons to the active site at the optimal flux and reduction potential to enable efficient catalysis.^5^ Of equal importance is how the internal electron transfer events integrate with external redox partners. A detailed description of the biophysical features of cofactors that exist at this interface is critical to fully understand the mechanisms of intramolecular electron transfer fundamental to driving a broad range of chemical reactions in biological systems.

Site-differentiated iron–sulfur clusters, where a canonical Cys residue is exchanged for another residue such as His, Asp, Glu, or Ser are prevalent in redox enzymes.^6,7^ They are often found as part of electron relays in proteins that perform unique chemical reactions, such as electron bifurcation where a pair of electrons are individually transferred from a single site down spatially and energetically separated pathways,^8–10^ DNA binding and regulation of gene expression,^11,12^ and the catalytic activation of small molecules (*e.g.*, H_2_).^13–15^ Studies have shown that site-differentiated coordination of iron–sulfur clusters can result in changes to the redox potential (*E*_m_), geometry, and electronic properties. These properties contribute to function in electron transfer embodied in the Marcus theory for non-adiabatic electron transfer (eqn (1)).^16^ The electron transfer rate constant (*k*_ET_) is determined by the tunneling matrix element |*T*_DA_^2^|, reorganization energy (*λ*), the total Gibbs free energy change (Δ*G*^0^) for the electron transfer reaction, and temperature (*T*).1
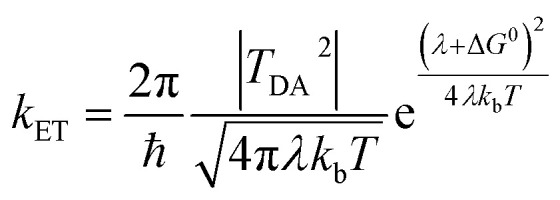


In this context, site differentiation of iron–sulfur clusters can be envisioned as a means to control electron transfer by tuning of Δ*G*^0^ (*i.e.*, *E*_m_), the density of electronic states |*T*_DA_^2^| (*i.e.*, geometry and bonding effects on spin states and spin pairing) and/or *λ* (*i.e.*, solvation).

**Fig. 1:**
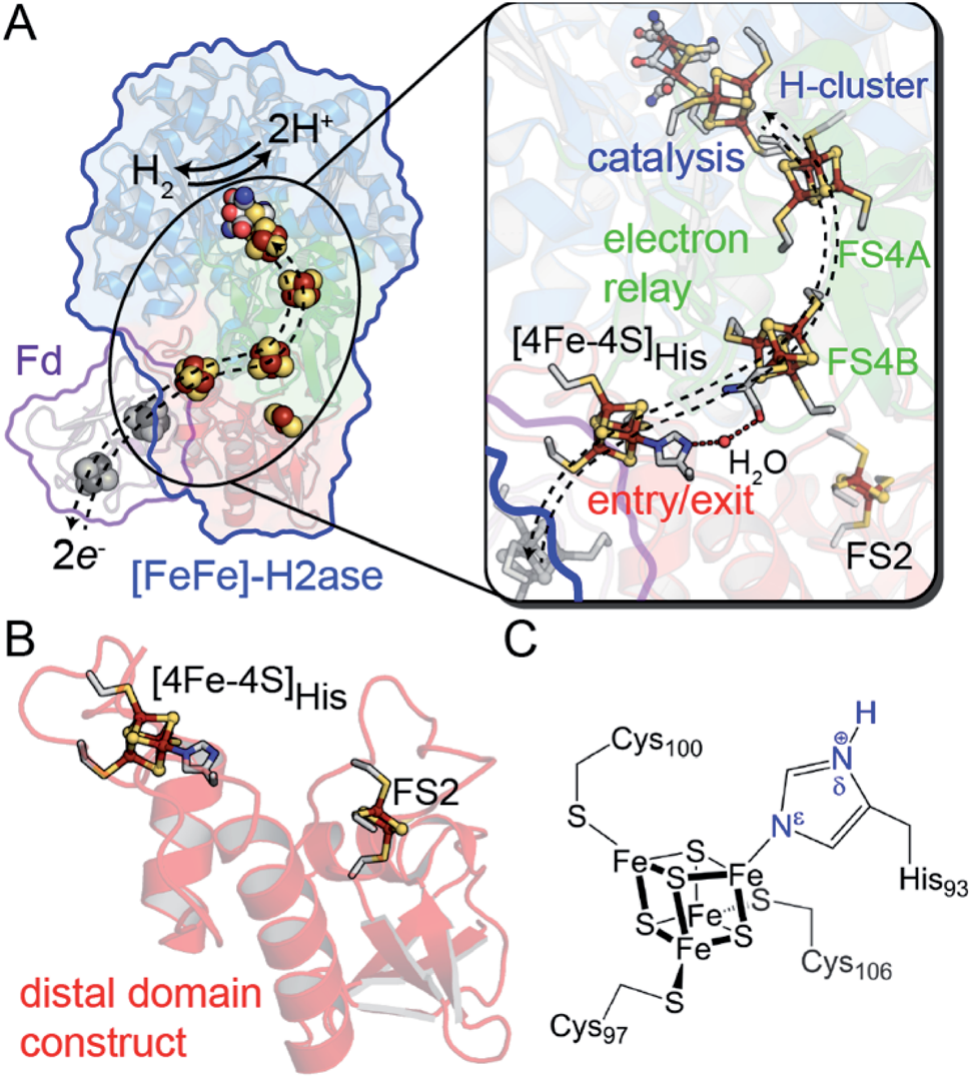
(A) Structural depiction of CpI [FeFe]-hydrogenase (PDB 3C8Y)^17^ and its redox partner ferredoxin (PDB 1CLF),^18^ which mediates electron transfer to CpI/CaI. Right, magnification of the electron transfer pathway (dashed lines) that consists of an entry-exit point at the surface localized [4Fe–4S]His cluster (red domain) connected by a relay through several [4Fe–4S] F-clusters (green domain, FS4A and FS4B) to the catalytic H-cluster (blue domain). (B) Representation of the distal domain construct used in this study. (C) Atomic detail of the distal [4Fe–4S]His cluster showing Cys and His coordination (amino acid numbering from CaI). Abbreviations: Fd, ferredoxin; FS4, [4Fe–4S]; FS2, [2Fe–2S].

One example of site differentiation is found in the [FeFe]-hydrogenase from *Clostridium pasteurianum* (CpI) and the structurally homologous enzyme from *C. acetobutylicum* (CaI). These enzymes catalyze H_2_ activation at a conserved organometallic, iron–sulfur cluster, or H-cluster, that is integrated with a conduit of iron–sulfur clusters, termed the F-clusters. F-clusters function in the transfer of electrons between external donor–acceptor molecules and the H-cluster during catalysis. The distal end of the conduit branches, ending at a site-differentiated [4Fe–4S] cluster with 3Cys1His ligation ([4Fe–4S]His) or a [2Fe–2S] cluster (Fig. 1).^15,19^ These clusters function in mediating electron transfer with external redox donors, for example ferredoxin,^15,20,21^ that are steered along a pathway formed by several [4Fe–4S] clusters within the protein to and from the H-cluster. Therefore, the entry-exit site for electron transfer has been proposed to involve either one of the two distal clusters, [4Fe–4S]His or [2Fe–2S] (Fig. 1B).^15,19,22,23^ In support of the electron transfer model with the [4Fe–4S]His cluster, molecular docking studies of CpI with Cp ferredoxin identified the [4Fe–4S]His as the binding site for interfacial electron transfer.^19^ Similar electrostatic models of ferredoxin binding with CaI places the distal [4Fe–4S]His cluster adjacent to a ferredoxin [4Fe–4S] cluster, which altogether suggest that the surface surrounding [4Fe–4S]His is optimized for binding ferredoxin to facilitate electron exchange.^24^

In this study, we demonstrate that site-differentiation of the surface localized [4Fe–4S]His cluster (shown in detail in Fig. 1C) of [FeFe]-hydrogenases CaI and CpI tunes the electronic structure and surrounding environment to facilitate electron transfer. A comparison of wild-type CaI to a His-to-Cys variant where the [4Fe–4S] cluster His-ligand was substituted by Cys, demonstrates that the change in primary coordination causes profound change in the surrounding molecular orbital configuration and electronic properties of the distal [4Fe–4S] cluster, with only modest changes in reduction potential. Catalytic activities measured with native electron mediators show higher values for native CaI *versus* the His-to-Cys variant. In conjunction with computational molecular orbital analyses, the results demonstrate that site-differentiation of iron–sulfur clusters modulates their underlying electronic properties for steering electrons through electron transfer pathways. Finally, we contextualize our findings within the Marcus theory framework to highlight how differential ligation may present a holistic paradigm for affecting enhanced electron transport by simultaneously impacting free energies, orbital overlaps, and solvent reorganization energies.

## Results

### Design of CaI distal domain constructs

To specifically probe the biophysical properties of the distal [4Fe–4S]His cluster of CaI free from contributions by the other iron–sulfur clusters, a truncated construct was created that consisted of the protein fold around the distal [4Fe–4S]His and [2Fe–2S] clusters. The construct was composed of the first 128 N-terminal residues of CaI (hereafter referred to as distal domain, DDHis) and recombinantly expressed in *Escherichia coli* (Fig. 1B). Following purification and iron–sulfur cluster reconstitution, 6.2 ± 0.2 mol Fe/protein was determined, confirming complete incorporation of both iron–sulfur clusters. A variant of the distal domain (DDCys) wherein the His-ligand was substituted by Cys was also constructed, allowing for explicit determination of properties unique to His coordination. Again, a value of 6.1 ± 0.4 mol Fe/protein was determined, showing that the His residue is not explicitly required for proper incorporation of a [4Fe–4S] cluster in this site. The iron–sulfur clusters in both constructs were found to be highly stable once incorporated into the protein scaffold, *i.e.*, clusters remained intact indefinitely across a broad range of pH values (6.5–10.5) under anaerobic conditions. Both DDHis and DDCys displayed typical ligand-to-metal charge transfer bands in their absorption spectra characteristic of oxidized iron–sulfur clusters, which diminished in intensity following reduction by sodium dithionite (Fig. S1†).

### Raman spectroscopy of the [4Fe–4S]His cluster

His coordination of [4Fe–4S]His in the reconstituted DDHis was confirmed by Raman spectroscopy, which displayed a unique band at 280 cm^−1^, absent in the DDCys spectra, that is located in the region where Fe–N stretching is expected to have a fundamental frequency (220–280 cm^−1^) in spectra of iron–sulfur cluster proteins (Fig. S2†).^25^ The remaining observed bands correspond well with Fe–S stretching modes (terminal and bridging) and are consistent with other [4Fe–4S] and [2Fe–2S] cluster containing proteins.^25,26^ When DDHis was poised at pH 5.9, below the His p*K*_a1_ (average of 6.6 in protein environments)^27^ the entire [4Fe–4S] cluster was partially degraded as evidenced by the attenuation of the Fe–S bands (Fig. S3†), and loss of intensity of the charge transfer absorption bands (data not shown). In contrast, DDCys did not show any significant changes with pH. These results are consistent with protonation of His Nε (Fig. S4†) and disruption of the Fe-Nε ligation, leading to cluster deterioration. This model for the pH sensitivity of DDHis also explains why there is a lack of a low pH effect on the stability of DDCys.

### Determination of [4Fe–4S] cluster redox potentials

**Fig. 2:**
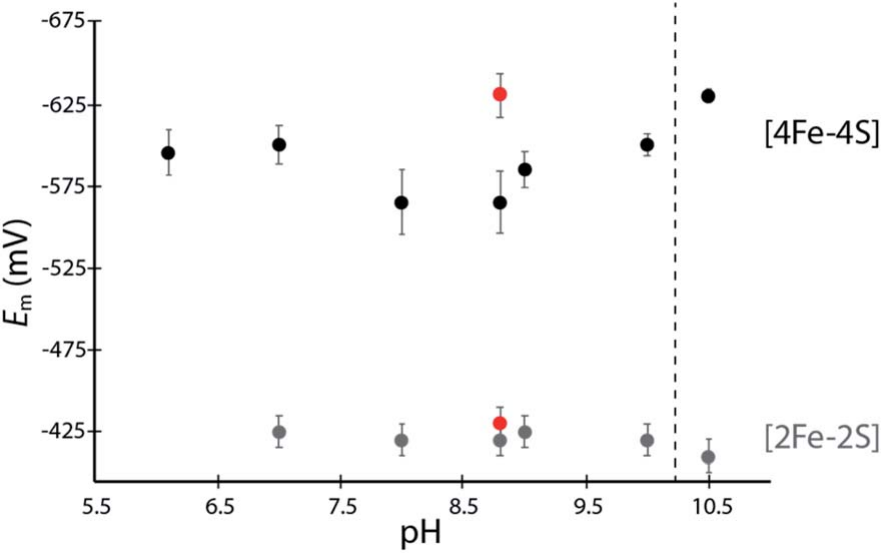
Scatter plot of measured *E*_m_ values for DDHis (black dots) and DDCys (red dots) iron–sulfur clusters at various solution pH values. Error bars represent standard deviation from at least 5 scans.

The [4Fe–4S]His cluster has been implicated to possess a low reduction potential (*E*_m_ <−450 mV *vs.* SHE), although it has not been directly measured.^19^ To quantify the low reduction potentials of these [4Fe–4S] clusters, we employed square wave voltammetry (SWV) for both DDHis and DDCys (Fig. 2 and S5†). Both constructs gave a similar *E*_m,8.8_ value of −420 mV for the [2Fe–2S] cluster, relatively consistent with previously published values for CpI (−360 mV to −404 mV).^19,28^ The *E*_m_ of [4Fe–4S]His was −565 mV at pH 8.8, whereas substitution of His for Cys led to a *E*_m,DDCys_ = −630 mV at the same pH, 65 mV lower than the native cluster. For comparison, the *E*_H^+^/H_2__ couple is −520 mV *vs.* SHE at pH 8.8 and 1 atm H_2_. Therefore, coordination by His or Cys results in *E*_m_ that is favourable for transfer of electrons with *C. acetobutylicum* ferredoxin (*E*_m_ = −480 mV at pH 8.8) during H_2_ oxidation.^21^

A dependence of the DDHis [4Fe–4S]His reduction potential on pH was investigated owing to the possibility that the His N-ligand may undergo changes in protonation during reduction–oxidation and electron transfer. SWV was performed at pH values between 6 to 10.5 using buffers with similar compositions (Good's buffers sharing ethanesulfonic acid groups). No pH dependency was observed for DDHis between pH 6–10 (*E*_m_ values are the same within error), however notable changes in reduction potential were found when the solution pH was increased above 10 (Fig. 2). At pH 10.5, the *E*_m_ value of [4Fe–4S]His became like that of DDCys, −630 mV. This observation can be rationalized where a more basic solution weakens the H-bonding character of the Nδ, leading to deprotonation of DDHis Nδ at pH 10.5 (p*K*_a2_ > 10, Fig. S4†) because Nε is already deprotonated *via* ligation with Fe (*e.g.* Rieske center His p*K*_a2_ values are reported to be 7.5–11.9;^29,30^ and free imidazole p*K*_a2_ is 14.52)^31^ (Fig. 2). This would presumably lessen the electron withdrawing ability of His, and a lower [4Fe–4S]His reduction potential at pH 10.5 is consistent with this model by stabilizing the [4Fe–4S]^2+^ oxidation state.

### CaI reactivity

To determine whether altering the distal cluster *E*_m_ and spin properties affects the reactivity of CaI, a holoenzyme mutant was generated where His coordination of the distal [4Fe–4S] cluster was changed to Cys (H93C CaI). The WT and H93C CaI hydrogenases were investigated for H_2_ production and uptake activities using *C. acetobutylicum* ferredoxin (CaFd, gene ID CAC0303), the native electron donor/acceptor to CaI.^20^

**Fig. 3:**
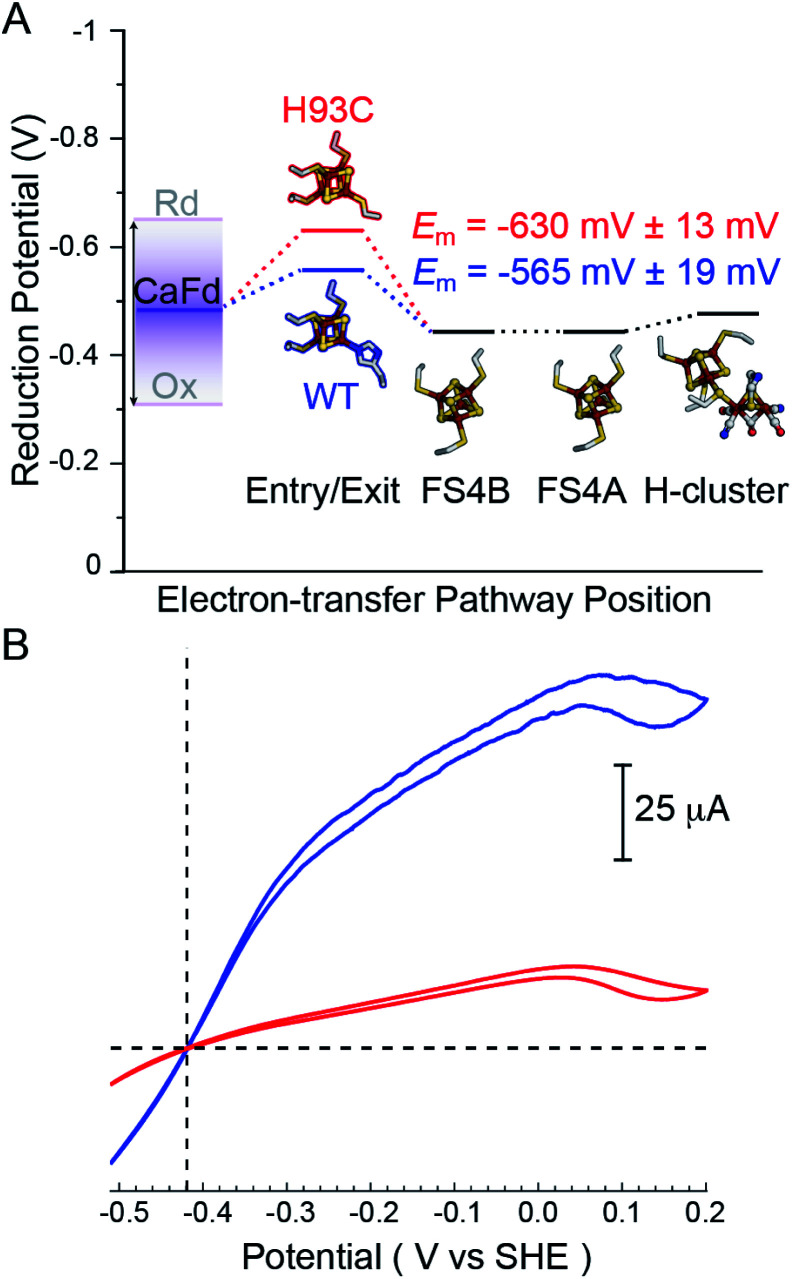
(A) Thermodynamic landscape for electron transfer between the catalytic H-cluster in CaI and its redox partner ferredoxin. The scheme shows *E*_m_ values for the distal [4Fe–4S] cluster of WT CaI (blue) and H93C CaI (red) obtained in this study, in relation to the *E*_m_ range for reduced (Rd) and oxidized (Ox) CaFd,^21^ the *E*_m's_ of F-cluster relay comprised of [4Fe–4S] clusters FS4B and FS4A, and the formal potential of the H-cluster at pH 8.8.^19^ (B) Electrocatalytic profiles of CaI WT (blue) and CaI H93C (red). Voltammograms were collected under 1 atm of H_2_ at a potential scan rate of 1 mV s^−1^. Other experimental conditions are pH 7.0, 25 °C and an electrode rotation rate of 3500 rpm. The dotted horizontal line shows zero current.

The H_2_ uptake and evolution activities were measured at pH 6 and 8.3 in both dye-linked (methylene blue, MB and methyl viologen, MV) and ferredoxin linked assays. In all conditions, the activities were significantly altered in the H93C CaI *versus* WT CaI. H_2_ production activity was reduced 3-fold and uptake activity was decreased 5-fold in H93C CaI (Table S1†). The activity effect was stronger for ferredoxin mediated reactions than for assays employing MB (H_2_ uptake) and/or reduced MV (Table S1†). CaFd has been hypothesized to bind to CaI within electron transfer distance of either the distal [4Fe–4S]His^19^ or [2Fe–2S] cluster.^23^ Interestingly, the *K*_M_ for CaFd was not significantly changed between WT and H93C CaI (1.15 ± 0.20 μM and 0.88 ± 0.15 μM, respectively, Fig. S6†). Thus, the lower CaFd-mediated rates demonstrate that a change in the unique coordination of the distal [4Fe–4S]His cluster from His-to-Cys affects the interfacial and/or intramolecular electron transfer process with H93C. This may be influenced by the slightly lower reduction potential of [4Fe–4S]Cys *versus* [4Fe–4S]His relative to CaFd (Fig. 3A), that changes the free-energy of interfacial electron transfer.

Free-energy effects on electron transfer in redox enzymes can be examined using protein film voltammetry. This technique enables access to a wide window of driving force (Δ*E*°), which complements the Fd assays to inform on electron transfer events over a broader range. In this case, the electrocatalytic profiles displayed similar overall shapes, however WT CaI was significantly steeper than H93C (Fig. 3, blue *versus* red). The more constant linear growth of H93C CaI indicates a limitation in electron transfer activity. Since the enzymes differ only in the coordination of the distal [4Fe–4S] cluster, the results are due to differences in interfacial electron exchange rates that arise from changes in the [4Fe–4S] cluster coordination.

### Electronic and magnetic features of [4Fe–4S]His

**Fig. 4:**
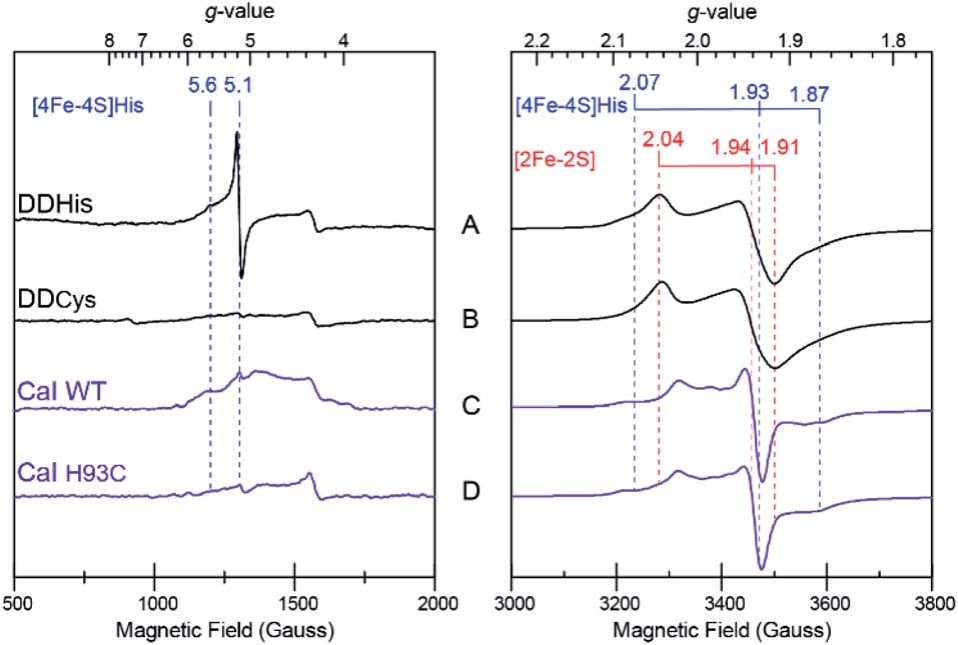
X-band EPR spectra of distal domain and CaI, with low-field region on left; high-field region, on right. (A) DDHis (230 μM) (B) DDCys (230 μM) (C), CaI WT (200 μM) (D) CaI H93C (200 μM). All samples were prepared under identical conditions and reduced with dithionite in pH 8.8 buffer. Signal assignments for the [4Fe–4S]His and [2Fe–2S] clusters are indicated. Measurement conditions: microwave frequency 9.39 GHz; modulation frequency, 100 kHz; modulation amplitude, 10 Gauss; microwave power, 1 mW (A and B), 10 mW (500–2000 Gauss C and D), 1 mW (3000–4000 Gauss C and D); sample temperature: 10 K.

In addition to driving force effects, the underlying electronic and magnetic properties of iron–sulfur clusters determine how they function in electron transfer. To define these properties of the [4Fe–4S]His cluster in CaI, X-band CW EPR on the DDHis and DDCys proteins were performed and used to resolve the more complex spectral properties of the WT and H93C full enzyme counterparts. When DDHis was reduced with dithionite, two *S* = ½ signals were identified and assigned to the [2Fe–2S] (*g* = 2.04, 1.94, 1.91) and [4Fe–4S]His (*g* = 2.07, 1.93, 1.87) clusters based on their unique temperature and power properties^32^ and agreement with prior EPR analysis of the homologous CpI protein^19^ and a CpI protein fragment containing only the [2Fe–2S] cluster^28^ (Fig. 4). The signal assigned to the [4Fe–4S]His cluster was detected between 5 and 20 K, whereas the signal assigned to the [2Fe–2S] cluster could be observed up to 60 K (Fig. S7†). Broadening of the spectrum was observed at low temperatures and is indicative of electron–electron spin coupling between the two clusters. In addition to the *S* = ½ signals in the *g* = 2 region, low-field resonances were observed including a predominant isotropic signal at *g* = 5.1 and a weaker, absorption peak at *g* = 5.6 (Fig. 4A). These displayed different temperature and power behaviour, indicating each is a unique signal (Fig. S8†). Both low-field resonances were largely attenuated in the DDCys mutant protein supporting their assignment to the [4Fe–4S]His cluster, while the *S* = ½ signals in the *g* = 2 region assigned to the [2Fe–2S] cluster remained largely unchanged (Fig. 4B). Similar low-field resonances were observed for WT CaI, albeit broadened and weaker in intensity (Fig. 4C). Like DDCys, the low-field resonances were almost completely attenuated in the H93C CaI variant (Fig. 4D). Conversely, the signal composition and intensity in the *g* = 2 region remained highly similar between WT and H93C. Spectral features of the [4Fe–4S]His signal identified in DDHis were also present in the WT spectrum, indicating its reduction. Compared to DDHis, the signal in the *g* = 2 region is more complex due to the additional FS4A and FS4B F-clusters which gives rise to strong intensity in the middle of the spectrum and is indicative of magnetic coupling among the clusters.^19^ Additionally in the WT and H93C, the H-cluster gives rise to a weak feature near *g* = 2.1 due to formation of its oxidized state (H_ox_) under the turnover conditions of the sample, yielding the well characterized *S* = ½ signal at *g* = 2.10, 2.04, 2.00.^33^ Overall, the similarity between WT and H93C spectra suggests minimal influence of the ligand alteration on the other *S* = ½ F-cluster and H-cluster signals. It is also noted that for all the spectra reported here, a small residual signal at *g* = 4.3 was observed and is thought to arise from a small Fe^3+^ impurity.

The low-field signals observed at *g* = 5.6 and 5.1 are most consistent with *S* > ½ spin states, and are similar to EPR signals that arise from half-integer Kramer systems in other [4Fe–4S] cluster containing proteins.^34–38^ In the highly homologous [FeFe]-hydrogenase from CpI, low-field resonances at *g* = 5.80 and 4.76 were also observed in reduced samples and assigned to an undefined *S* = 3/2 spin system.^33^ Similar low-field resonances at *g* = 5.70, 5.55, and 5.15 were also observed for the [FeFe]-hydrogenase from *Thermotoga maritima* and suggest the presence of multiple *S* > ½ species.^39^ As shown in Fig. 4, comparing the EPR spectrum of the low complexity DDHis protein to DDCys and His-to-Cys substituted H93C CaI variant enabled a definitive assignment of the low-field resonances to the [4Fe–4S]His cluster. Simulations of the *g* = 5.1 were used to assign it to a ±3/2 doublet of the *S* = 7/2 spin system with *E*/*D* = 0.125, where *D* and *E* are the respective axial and rhombic zero-field splitting parameters (Fig. S9†). This assignment is supported by rhombograms that predict an intense isotropic signal where all three *g*-values coincide at ∼*g* = 5 for *E*/*D* = 0.12 as is observed in low-field spectra of DDHis in Fig. 4A.^32^ Simulation of the weaker signal at *g* = 5.6 assigns it to the upper ±3/2 doublet of the *S* = 3/2 spin system with an *E*/*D* = 0.28. Notably, the observation of a mixed spin state in [4Fe–4S]His cluster (*e.g. S* = 1/2, 3/2, and 7/2 states), which is lost upon conversion to full Cys coordination, demonstrates that His coordination increases the overall density of states that are energetically accessible.^40^

The diminished and broadened signal intensity of the CaI EPR spectrum in the low-field region compared to DDHis suggests the *S* > ½ spin states may be sensitive to additional interactions not present in the truncated protein. One possibility is that inhomogeneous broadening of the *S* > ½ signals is due to a magnetic exchange interaction of the distal [4Fe–4S]His cluster with the F-cluster relay (*e.g.* FS4B), as has been shown for the observed *S* = ½ signals that are reflective of a reduced F-cluster relay.^19^ Evidence of magnetic interactions indicates long-range electronic interactions that extend throughout the F-cluster relay and includes the [4Fe–4S]His cluster. Together with the increased spin-states, the electronic properties of [4Fe–4S]His function to facilitate more reversible electron transport to and from the catalytic H-cluster compared to [4Fe–4S]Cys.

### Quantum mechanical calculations of the distal [4Fe–4S] cluster

Insights into the impacts of differential coordination on the electronic structure of the distal [4Fe–4S]His cluster were probed at the quantum mechanical level involving density functional theory using the broken-symmetry approach.^41,42^ In our calculations, we considered the primary coordination sphere truncated at the C_β_ carbon atom for the site-differentiated His-coordinated cubane structure in its unprotonated ([4Fe–4S]His) and protonated ([4Fe–4S]HisH) states, and compared it with the all Cys coordinated cubane structure ([4Fe–4S]Cys) (Fig. S10†). The presence of magnetic coupling amongst the Fe atoms of the cubane cluster results in three possible spin pairing combinations characterized by the specific oxidation states on the individual Fe atoms of the cluster. Previous studies focused on [4Fe–4S] clusters have exploited the symmetry of the all Cys coordinated clusters to consider only a single spin-pairing combination for their calculations.^43^ Because the site-differentiation of the ligands as well as their geometries in the protein results in asymmetry, that precludes this simplification. Therefore, we considered all three possible spin pairing combinations – ααββ, βαβα and αββα (Fig. S11†).

Redox potential calculations (Table S2†) for the clusters with the three possible ligand environments revealed a Cys-coordinated cluster redox potential of *E*° = −1622.16 mV (*vs.* theoretical, absolute SHE at pH = 0). This value is considerably more negative compared to both [4Fe–4S]His (*E*° = −1510.82 mV) and [4Fe–4S]HisH (*E*° = −1297.12 mV), where the protonated version of HisH has the most positive potential. This trend is in agreement with our experimental observations of DDHis at pH < 10 having a more positive *E*_m_ value than DDCys (Fig. 2) and is consistent with other recent theoretical estimates.^43^

**Fig. 5:**
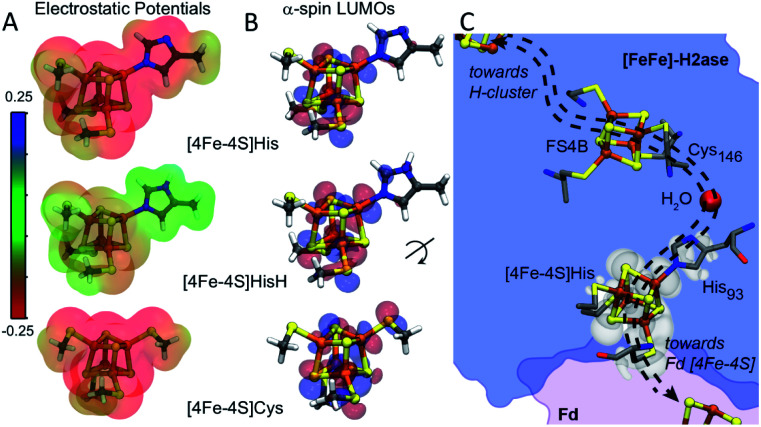
(A) Electrostatic potentials depicted on electron density iso-surfaces for each cluster system in the ααββ spin pairing combination in the oxidized state. Red regions show volumes of high negative charge while green regions show volumes of neutral charge. [4Fe–4S]HisH is observed to be much less negatively charged compared to [4Fe–4S]Cys. (B) Depictions of lowest unoccupied molecular orbitals (α-LUMOs) for the three clusters in the oxidized state, indicates regions with high probability for hosting the electron upon reduction. The red and blue delineate the wave-function as being either negative or positive, respectively. (C) The presence of HisH elongates the molecular orbitals of the [4Fe–4S], shown by superposition of the[4Fe–4S]HisH LUMO map (white shade) onto the CaFd/CaI electron transfer complex. Orientation of the electronic structure towards the coordinated water molecule imparts directionality to electron transport between CaFd and the accessory [4Fe–4S] cluster relay (shown as dashed arrows).

Amongst the three spin pairing combinations considered in this study, the ααββ combination presents the most plausible electronic configuration to mediate electron transport from the bound ferredoxin to the H-cluster since it couples the site of ferredoxin binding at the [4Fe–4S]His cluster (specifically Fe4) with the His coordinated iron (Fe3) atom (Fig. S11†). We thus analysed the impact of His coordination on the electrostatic potentials (ESP) and molecular orbitals (MOs) of the cubane structure for the ααββ spin-pairing combination. In Fig. 5A the electronegative and positive regions of the cluster are depicted on a surface representing the electronic density around the cluster and can be used to qualitatively interpret the relative electron accepting nature of the clusters in the three ligand environments. In the oxidized state of the cluster, the presence of highly negative ESP in Cys and His coordinated clusters makes it less energetically favourable to accept an electron. Alternatively, the more neutral ESPs in the HisH coordinated cluster means that it has a lower energetic penalty to accept an electron for reduction.

Fig. 5B illustrates the effect of changes in the site-differentiated coordinating residue on the lowest unoccupied molecular orbitals (LUMOs) of the distal [4Fe–4S] cluster. The LUMO is the lowest energy molecular orbital that does not host an electron and, in the oxidized state, can be considered as the most likely place for hosting the extra electron that would be received by the cluster upon reduction. In the comparison of MO's of [4Fe–4S]Cys to [4Fe–4S]His or [4Fe–4S]HisH, it is evident that the MO's for the [4Fe–4S]Cys extend only to the S-atom of the coordinating Cys residue. However, in the case of [4Fe–4S]His and [4Fe–4S]HisH, the MO's are observed on the Nδ and coordinating Nε atoms of the His imidazole ring, and thus extended closer to the nearby FS4B [4Fe–4S] cluster. Interestingly, there is a coordinated water in the crystal structure of CpI that bridges the His93 Nδ atom to the carbonyl carbon of Cys146, which coordinates to the adjacent [4Fe–4S] cluster. This water coordination is expected to be changed or be lost when His is replaced with Cys. Hence, our MO analysis for the ααββ spin pairing combination of [4Fe–4S]His suggests that His coordination creates a tunnelling network to direct electron transport to and from the neighbouring [4Fe–4S] cluster in the F-cluster relay (Fig. 5C).

## Discussion

The effect of site-differentiation on [4Fe–4S] clusters, specifically where a mixture of Cys and His coordinating residues is observed, has been shown to have differential effects on the *E*_m_ value. For example, in the case of the His coordinated [4Fe–4S] cluster in the [NiFe]-hydrogenase from *Desulfovibrio fructosovorans* a Cys substitution leads to a slight positive shift in *E*_m_.^44^ For CaI we demonstrated that the substitution of His for Cys leads to a small negative shift in *E*_m_ of ∼65 mV. In the context of non-adiabatic electron transfer (eqn (1)) there are slightly smaller barriers in the free-energy (Δ*G*°) profile for electron transfer with ferredoxin and the F-cluster relay in [4Fe–4S]His *versus* [4Fe–4S]Cys CaI, that favours reversibility.

The EPR measurements and computational modelling of CaI [4Fe–4S]His cluster demonstrate that His coordination also creates a uniquely tuned electronic structure of the cluster compared to all Cys coordination which impacts |*T*_DA_|. The LUMO map, which defines where electrons can localize on the [4Fe–4S] cluster, is less symmetric in [4Fe–4S]His with more of the LUMO residing in the space between CaFd and the [4Fe–4S]His cluster to enable electron exchange between the two species. The imidazole ring of [4Fe–4S]His also serves to extend the MO network for electron tunnelling closer to the nearby [4Fe–4S] cluster and includes a coordinated water. Similar extended MO networks and presence of coordinated water have been shown to greatly enhance electronic coupling and electron transfer rates between iron–sulfur clusters in complex I.^45^ In addition, the [4Fe–4S]His cluster has a higher density of electronic states compared to [4Fe–4S]Cys, which are energetically accessible during the reduction–oxidation events of electron transfer.^40^ This was observed from a stabilized *S* = 7/2 spin system that is lost with a change to Cys. A more symmetric spin localization ([4Fe–4S]Cys) *versus* the less symmetric, more delocalized ([4Fe–4S]His) cluster has been proposed to impact rate constants with donor–acceptors through spin exchange interactions.^46^

Although solvent reorganization energies (*λ*) were not calculated for [4Fe–4S]His or [4Fe–4S]Cys, a recent computational study on a His-ligated distal FeS cluster in a [NiFe]-hydrogenase revealed that His-ligation did not result in significant changes to *λ*.^47^

## Conclusion

We have demonstrated that His ligation of the distal [4Fe–4S] cluster in CaI tunes the cluster's reduction potential, bonding and electronic properties that in turn tune the reversibility of intermolecular and intramolecular electron transfer for coupling to reversible catalytic H_2_ activation.^44,47,48^ The results presented here broadly inform on the functional effects of site-differentiation, and how it may be a common approach that biology employs to control electron transfer reactions within enzymes and with electron mediators. It is anticipated that this is a more general mechanism to optimize kinetics, directionality, and coupling of electron transfer in complex energy transduction systems.^5^ For CaI, the unique coordination of the [4Fe–4S]His cluster acts to integrate the electronic structure and physical environments to enable facile electron exchange, an advantageous property for electron transfer with ferredoxin.

## Data availability

Experimental and computational data files that are not included in the ESI† will be made available upon request by an email to the corresponding author.

## Author contributions

The manuscript was written through contributions of all authors. All authors have given approval to the final version of the manuscript.

## Conflicts of interest

There are no conflicts to declare.

## Supplementary Material

SC-013-D1SC07120C-s001
