# Supplementary material for: A site-differentiated [4Fe–4S] cluster controls electron transfer reactivity of Clostridium acetobutylicum [FeFe]-hydrogenase I
Source: Chem Sci. 2022 Mar 25;13(16):4581–8. doi: 10.1039/d1sc07120c (PMC9019909; doi:10.1039/d1sc07120c)
Supplement: SC-013-D1SC07120C-s001 [file SC-013-D1SC07120C-s001.pdf]

Electronic Supplementary Material (ESI) for Chemical Science.
This journal is © The Royal Society of Chemistry 2022

Supporting Information

A Site-Differentiated [4Fe-4S] Cluster Controls Electron Transfer Reactivity of *Clostridium acetobutylicum* [FeFe]-hydrogenase I

Carolyn E. Lubner[a], Jacob H. Artz[a], David W. Mulder[a], Aisha Oza[a], Rachel J. Ward[b], S. Garrett Williams[c,d], Anne K. Jones[c], John W. Peters[e], Ivan I. Smalyukh[b,f], Vivek S. Bharadwaj[a], and Paul W. King[a,f*]

[a] *National Renewable Energy Laboratory, Golden, Colorado, USA.*
[b] *Department of Physics, University of Colorado Boulder, Boulder, Colorado USA.*
[c] *School of Molecular Sciences, Arizona State University, Tempe, Arizona, USA.*
[d] *Sandia National Laboratories, Albuquerque, New Mexico, USA.*
[e] *Institute of Biological Chemistry, Washington State University, Pullman, Washington, USA.*
[f] *Renewable and Sustainable Energy Institute, National Renewable Energy Laboratory and University of Colorado Boulder, Boulder, Colorado USA.*

EXPERIMENTAL METHODS

Protein Expression and Purification. [FeFe]-hydrogenase CaI wild-type and the site-differentiated iron-sulfur cluster ligand mutant (H93C) were heterologously expressed in *E. coli* by co-transformation of the BL21(DE3) Rosetta-2 cell line with structural and maturase gene constructs (pCaAE, pCaFG) as previously described.[1] Truncated mutants of CaI (distal domains of WT and H93C) containing only the first 128 residues were synthesized with a C-terminal Strep tag by GenScript and cloned into pCDFDuet-1. Heterologous protein expression was performed using the BL21(DE3) Rosetta-2 *E. coli* strain (Novagen). Fresh transformants were inoculated into 5 mL Terrific Broth media (EMD Millipore) with appropriate antibiotics and grown overnight at 37 °C and 250 rpm shaking. The next day, the cells were washed and resuspended in fresh TB media and used to inoculate 50 mL TB (1:100). The culture was grown at 37 °C and 250 rpm shaking until OD600 of 0.4. 1L TB media was inoculated 1:100 with the pre-culture and grown until OD600 of 0.85 at which point it was induced with IPTG (1.5 mM) and supplemented with ferric ammonium citrate (2.5 mM) and continued to shake at 37 °C for 45 min. Cultures were then additionally supplemented with glucose (0.5%), sodium fumarate (10 mM), and L-cysteine (1 mM) and made anaerobic by sparging with Ar gas at room temperature overnight. Cultures were then harvested anaerobically, washed once with Buffer A (150 mM HEPES pH 8.8, 200 mM NaCl, 5% glycerol) and frozen at -80 °C until further use. Pellets were thawed and diluted with lysis buffer containing 20 mg lysozyme, 30 uL Pierce universal nuclease, 2 mM DTT and 1 tablet EDTA-free protease inhibitor cocktail (Roche Life Sciences), followed by passing through a microfluidizer 12 times. Broken cells were subjected to ultracentrifugation at 45 K rpm for 1 h at 4 °C to pellet the insoluble fraction. Supernatant was loaded onto an affinity column (StrepTactin Superflow high capacity resin, IBA packed into a column for FPLC (AKTA)) inside an MBraun anaerobic box. Highly pure protein was eluted from the washed column with 2.5 mM desthiobiotin. Purity was assessed by SDS-PAGE and protein concentration was determined using Bradford assay (Bio-Rad). Iron-sulfur clusters were reconstituted into the protein by incubation with 6 molar excess of ferrous ammonium sulfate, sodium sulfide and 2-mercaptoethanol at 4 °C overnight under anaerobic conditions. Reconstituted protein was then washed several times with Buffer A and concentrated by ultrafiltration (Amicon stirred cell) and followed by a final pass over PD-10 desalting column equilibrated with Buffer A to remove any residual unbound iron and sulfide. Iron analysis was performed as previously described.[2, 3] Purified, reconstituted protein was handled under strictly anaerobic conditions.

CaFd (CA_C0303 with a C-terminal Strep tag synthesized by GenScript and cloned into pCDFDuet-1) was expressed using the BL21(DE3) Rosetta-2 E. coli strain (Novagen). Fresh colonies were used to inoculate a 150 mL overnight culture, of which 4 mL/L were used to inoculate the expression media (LB). Cultures were grown at 37 °C and 250 RPM to O.D. 0.3, then induced with 1 mM IPTG and sparged with Ar. After 4 hours the cell pellet was harvested anaerobically, lysed and centrifuged as done for CaI. Purification was done over Strep XT-HC resin (IBA) and eluted using 5 mM biotin.

Activity Assays. Dye-linked assays for H_2 production or oxidation were performed with methyl viologen (MV) and methylene blue (MB), respectively, according to previously reported methods.[4] For Fd-linked H_2 production assays, Fd was first reduced with 10 mM DT and then buffer-exchanged to remove excess reductant. 100-fold molar excess of Fd was injected into a sealed serum vial with H2ase to initiate the reaction, and H_2 production was measured via GC. For H_2 oxidation, 100-fold molar excess of Fd was added to a sealed cuvette with H2ase under 100% H_2 atmosphere, and the reduction of Fd was monitored at 390 nm.

EPR Spectroscopy. Samples were prepared as described above and with the addition of the reductant, sodium dithionite, prepared in either the sample buffer (33 mM) or in 1M glycine pH 10 buffer (33 mM), as needed to adjust the solution potential. When 1M glycine pH 10 buffer was used, the solution pH was subsequently measured to account for small differences by addition of the strong base. The corrected pH values are reported where appropriate.

X-band CW EPR spectra were collected on a Bruker E-500 spectrometer equipped with a SHQ resonator, an in-cavity cryogen free VT system (ColdEdge Technologies) and MercuryiTC temperature controller (Oxford). To avoid power-saturation, low-field and high-field regions were collected separately. Temperature and power saturation experiments were carried out between 3K – 80K and 0.2 µW – 100 mW, respectively. Baselines were manually subtracted using the OriginPro software package. EPR simulations were carried out in MatLab using the EasySpin package and 'esfit' fitting function.[5]

Electrochemistry. Protein film electrochemistry (cyclic voltammetry) was performed in a nitrogen-filled anaerobic glovebox (MBraun Atmospheres). Electrochemical experiments were performed in a water-jacketed, temperature-controlled electrochemical cell using a PG-Stat 128N Autolab electrochemical analyzer controlled by GPES software. A three-electrode setup was utilized with a platinum wire counter electrode, a saturated Ag/AgCl reference electrode, and a custom 25π mm2 pyrolytic graphite edge (PGE, Minteq) working electrode. The electrode was rotated by an AFMSRCE Series Rotator (Pine Instrument Co.) Potentials were corrected to the standard hydrogen electrode (SHE) (Ag/Ag^+ + 0.197 = SHE). Protein films were produced by spotting 5-10 µL of protein solution onto the surface of the PGE electrode, incubating for 60 seconds at room temperature in the glovebox, and drying with nitrogen gas. Electrochemical

experiments employed 150 mM Goods buffers comprising PIPES or HEPES with 200 mM sodium chloride as a supporting electrolyte and five percent glycerol. Hydrogen was sparged into the electrochemical cell above the surface of the buffer solution at 1 atm as the electrode was rotated at 3,500 rpm.

Square wave voltammetry experiments were performed inside an MBraun anaerobic box, using a pyrolytic graphite edge working electrode (0.2 cm^2 surface area, Pine), an Ag/AgCl reference electrode, and a Pt wire counter electrode. The working electrode was prepared by polishing with an aqueous slurry of 1 µm alumina on a diamond polishing pad (BASi), rinsing with water, and further polishing with 0.05 µm alumina slurry on a microcloth pad (Buehler) and thorough rinsing with water.

Samples at different pH points were prepared by buffer exchange using a 10 kD cutoff spin column (repeated four times) with the desired buffer: PIPES (pH 6.1), HEPES (pH 7, 8, 8.8), CHES (pH 9, 10), and CAPS (pH 10.5). All buffers contained 150 mM buffer, 200 mM NaCl, and 5% glycerol.

Square wave voltammetry measurements employed a step potential of 1 mV, an amplitude of 25 mV, and frequency of 5 Hz. The experiments were controlled by a CH Instruments potentiostat (Model 660C). The measured reduction potentials were the average of 5-15 scans, each with fresh protein. Due to the negative charge of the protein, a drop method was used whereby the working electrode was operated upside down, a 50 µL drop of protein (345 µM) was placed on its surface, with reference and counter electrodes placed in the drop. This method was employed for all pH points except pH 6.1 and 7 since the protein possesses a positive charge due to its pI of 7.5. At these pH's, typical protein film methods were used. The differing immobilization strategies may be responsible for the slight differences in measured values, however values for all samples under pH 10.5 were within error and essentially the same. Scans of buffer only were taken for subtraction of buffer background in the protein experiments. Representative traces are shown in Figure S4.

Raman Spectroscopy. Samples were prepared for Raman spectroscopy by loading 7-10 µL of protein (345 µM) or buffer sample into rectangular 200 µm x 2 mm capillary tubes, and then sealing the ends of the capillary tubes with epoxy. Samples were loaded under nitrogen in an inflatable glovebox and held at −80 °C before and after loading to prevent exposure to oxygen. The loaded capillaries were then mounted on a custom-built sample plate for an Instec cooling stage and held at -85 °C for collection of Raman spectra using a setup described in detail elsewhere.[6] Raman spectra were collected using a 532 nm excitation beam and 60x oil-immersion objective lens. Spectra were collected at low power by accumulating 2000 x 0.2 s exposures at different sample positions along the capillary for a total integration time of ~7 min per spectrum. 10-20 repetitions of such accumulated spectra were collected for each sample. These spectra were then averaged and smoothed by adjacent averaging. Resulting protein and buffer spectra were compared to distinguish active Raman peaks unique to the protein samples.

COMPUTATIONAL METHODS

Molecular Model. The initial geometry for the histidine-ligated 4Fe-4S cluster was obtained from the crystal structure (PDB ID: 3c8y) of CpI hydrogenase, which has 71.3% identity with CaI hydrogenase.[7] The [4Fe-4S]His cluster was modeled with truncated ligands, methanethiol and 4-methylimidazole to represent cysteine ([4Fe-4S]Cys) and histidine ([4Fe-4S]His) respectively. [4Fe-4S]His was modelled in both its protonated state ([4Fe-4S]HisH) and unprotonated state ([4Fe-4S]His). The [4Fe-4S]Cys cubane structure was created by modifying the histidine ligand of the [4Fe-4S]His cluster geometry. Figure S10 shows the initial geometries for the three cubane structures considered in this study.

The four Fe atoms in the cluster are defined as Fe_1, Fe_2, Fe_3, and Fe_4 with Fe_3 being the site for differentiated ligand coordination. For the oxidized $[Fe_4S_4]^{2+}$ cluster, two Fe atoms must have a charge of 2+ and the other two must have a charge of 3+. There are three distinct spin pairings to consider for the anti-ferromagnetically coupled states, $[Fe_1^bFe_2^aFe_3^bFe_4^a]$, $[Fe_1^aFe_2^aFe_3^bFe_4^b]$, and $[Fe_1^aFe_2^bFe_3^bFe_4^a]$ where a and b represent the spins of the unpaired electrons of each Fe atom. We consider one reduced state from each of the spin-pairing combinations in which the atom being reduced is ferromagnetically coupled to Fe_3 and its corresponding oxidized states.

Broken-Symmetry Density Functional Theory (BS-DFT). The consideration of anti-ferromagnetic coupling in the [4Fe-4S] cluster necessitates the use of broken-symmetry DFT calculations which were all carried out using the Gaussian 16 program.[8] All calculations were performed with the unrestricted (U) BP86 functional supplemented with Grimme et al.'s D3 empirical diffuse function.[9-11] The def2TZVP/TZVPfit basis set/density fitting set were used.[12-15] SCRF conductor-like polarizable continuum model (C-PCM) was implemented with the solvent 2-NitroPropane (e = 25.654).[16, 17] This dielectric constant was chosen to resemble the environment of the [4Fe-4S]His cluster residing near the surface of the hydrogenase.[18]

The model system was separated into fragments to allow for a BS-DFT approach with the 4Fe atoms, $4S^{2-}$ and the 4 ligands specified as individual fragments. For the oxidized state, the overall charge on the cluster is assigned as -2. The QM workflow adopted to perform the BS-DFT calculations is illustrated in Figure S11. The high spin state (S=18/2) calculations for the oxidized cluster consisted of a geometry optimization for the ferromagnetically aligned Fe atoms, a frequency calculation, and a Hirshfeld population analysis.[19-21] The stability of the resulting wavefunction was probed and then that geometry was passed into the guess for the low spin state (S=0) which is achieved by flipping the spins on two Fe sites (one Fe^{2+} and one Fe^{3+}). The low spin geometry is optimized, a frequency calculation, single point energy, stability and population analyses follow. The reduced system is defined by an electron being added to the system, changing the overall charge of the cluster to -3. This results in high spin and low spin state multiplicities of S=17/2 and S=1/2 respectively, with the complexes being generated and studied the same way for the reduced state as the oxidized.

Optimized geometries, molecular orbitals and electrostatic potential surfaces for the clusters were visualized using Visual Molecular Dynamics (VMD) software.[22] Gaussian's cubegen function was used to generate electrostatic potential plots and molecular orbitals. To calculate redox potentials, we consider six proximal reductions. We define proximal reductions as reductions in which the Fe^{3+} atom reduced is part of the anti-ferromagnetic couple involving differentially ligated Fe_3. We consider the average of six reductions in which the atom reduced is not Fe_3, but rather the Fe atom coupled to it. The redox potentials are calculated as follows and are referenced to the theoretical absolute reduction potential of the standard hydrogen electrode at pH=0.[23]

$$E_{red} = \frac{-(G_{red} - G_{ox})}{nF} - 4.535\ V \quad (1)$$

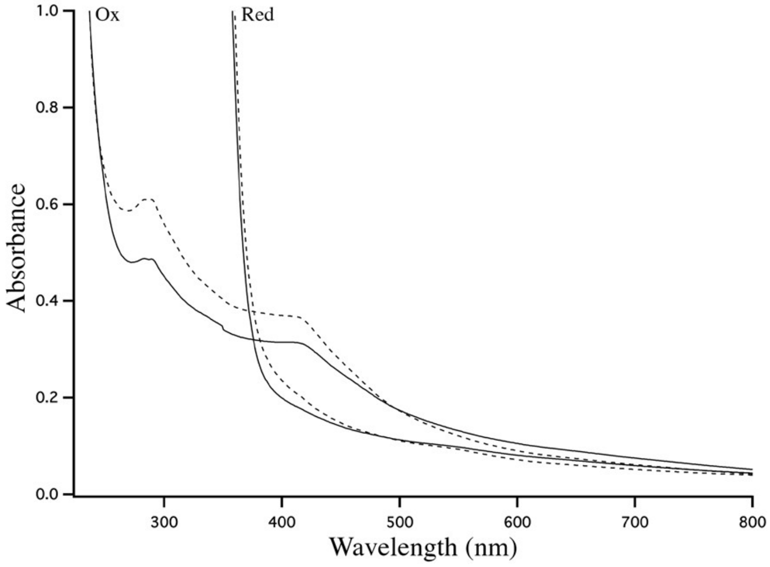

Figure S1. UV/Vis absorption spectra of oxidized and dithionite-reduced DDHis (solid line) and DDCys (dashed line). The absorbance at 420 nm, characteristic of Fe-S charge transfer bands in iron-sulfur clusters, disappears upon reduction with sodium dithionite, as expected. Identical features are observed for both constructs.

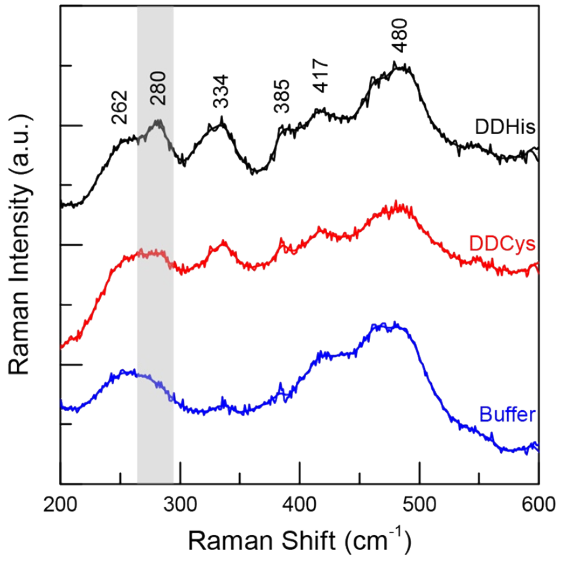

Figure S2. Raman spectroscopy of DDHis (black) and DDCys (red) proteins at pH 8.8 (345 µM). The unique band at 280 cm^{-1} in DDHis is attributed to the Fe-N bond arising from His ligation (grey bar). This feature is absent in the DDCys due to replacement of the Fe-N bond with an Fe-S bond. Buffer background spectra (blue).

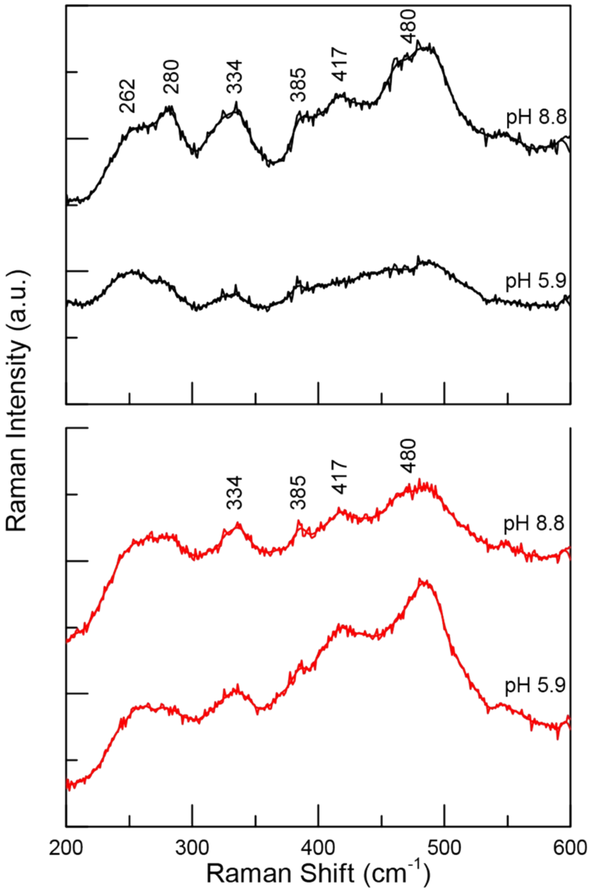

Figure S3. Raman spectroscopy of DDHis (top panel, black) and DDCys (bottom panel, red) proteins at pH 8.8 and 5.9. Fe-S and Fe-N bands decrease in intensity in DDHis due to deterioration of [4Fe-4S]His. These spectral changes are not observed for DDCys.

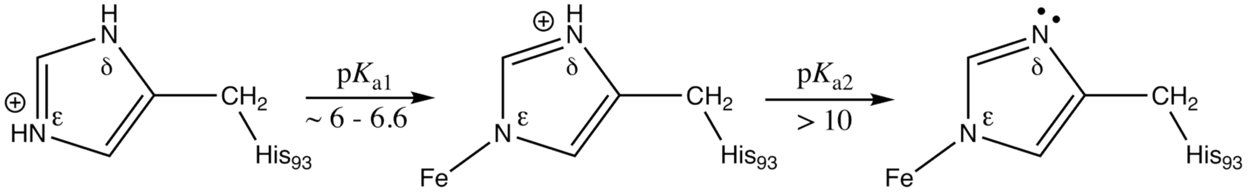

Figure S4. Changes in His protonation as a function of pH. Below pK_{a1}, iron ligation is less stable as protonation of Nε is favored, and results in cluster destabilization in DDHis. At physiological pH, the Fe-Nε bond is stabilized due to the electron withdrawing nature of the His ring. Above pK_{a2}, the Fe-His bond becomes more like Fe-Cys due to deprotonation of Nδ.

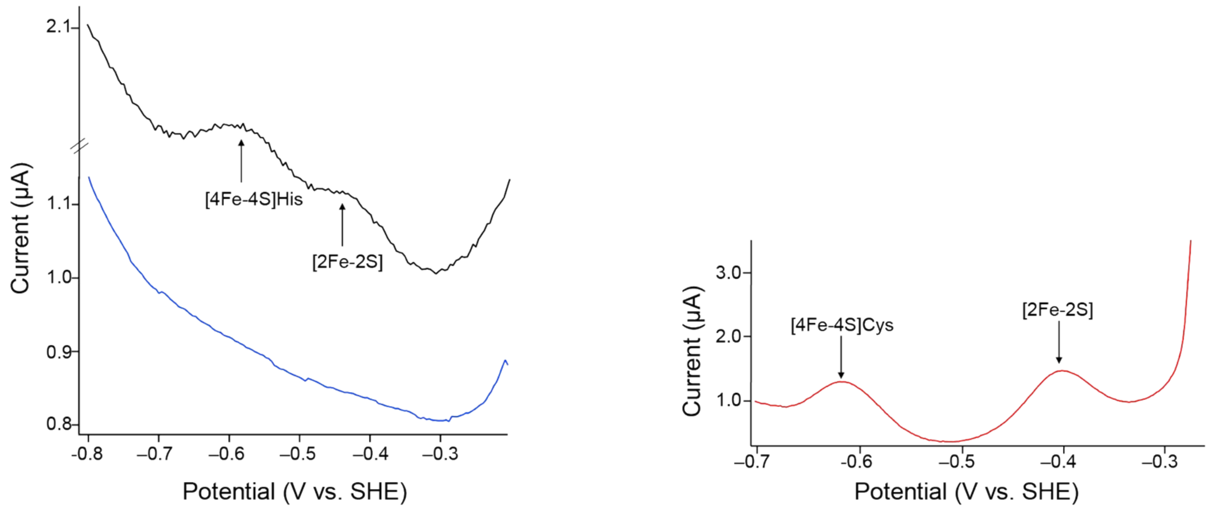

Figure S5. Representative square wave voltammograms for DDHis (left, buffer background in blue, protein in black) and DDCys (right, background subtracted protein in red).

Table S1. Biochemical H_2 production and uptake reactions for CaI WT and CaI H93C with electron mediators.

Enzyme and Mediator	H_2 Production $H_2ase + 2H^+ + 2Fd_{red} \rightleftharpoons H_2 + 2Fd_{ox}$ (µmol min^{-1} mg^{-1})		H_2 Uptake $H_2ase + H_2 + Fd_{ox} \rightleftharpoons 2H^+ + 2Fd_{red}$ (µmol min^{-1} mg^{-1})	
	**pH 8.3**	**pH 6**	**pH 8.3**	**pH 6**
WT CaI + MV/MB[1]	600 ± 60	ND	4559 ± 1354	6741± 498
H93C CaI + MV/MB[1]	428 ± 43	ND	2560 ± 768	4176 ± 859
WT CaI + CaFd[2]	300 ± 35	14 ± 2	10400 ± 200	1780 ± 283
H93C CaI + CaFd[2]	100 ± 50	8 ± 2	2050 ± 150	269 ± 21

[1] MV, methyl viologen (5 mM final concentration) used for H_2 production assays measured via GC; MB, methylene blue used for H_2 uptake assays, with reduction of MB monitored at 600 nm.

[2] 100-fold molar excess of reduced CaFd:CaI was utilized for H_2 production assays measured via GC, 100-fold molar excess of oxidized CaFd:CaI was utilized under 100% H_2 atmosphere for H_2 oxidation assays, with reduction of CaFd monitored at 390 nm.

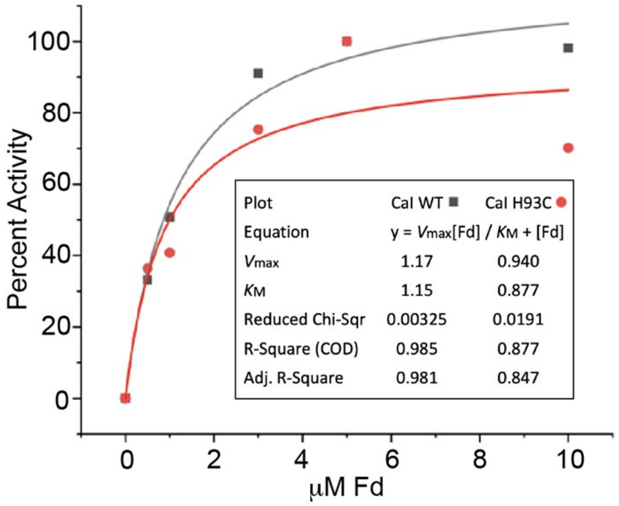

Figure S6. Michaelis-Menten reaction kinetics for the H_2 evolution activity rate of CaI WT and CaI H93C as a function of ferredoxin concentration. Ferredoxin $K_{m's}$ were determined from fits to the Michaelis-Menten equation indicated in the inset for CaI WT (black trace) and CaI H93C (red trace).

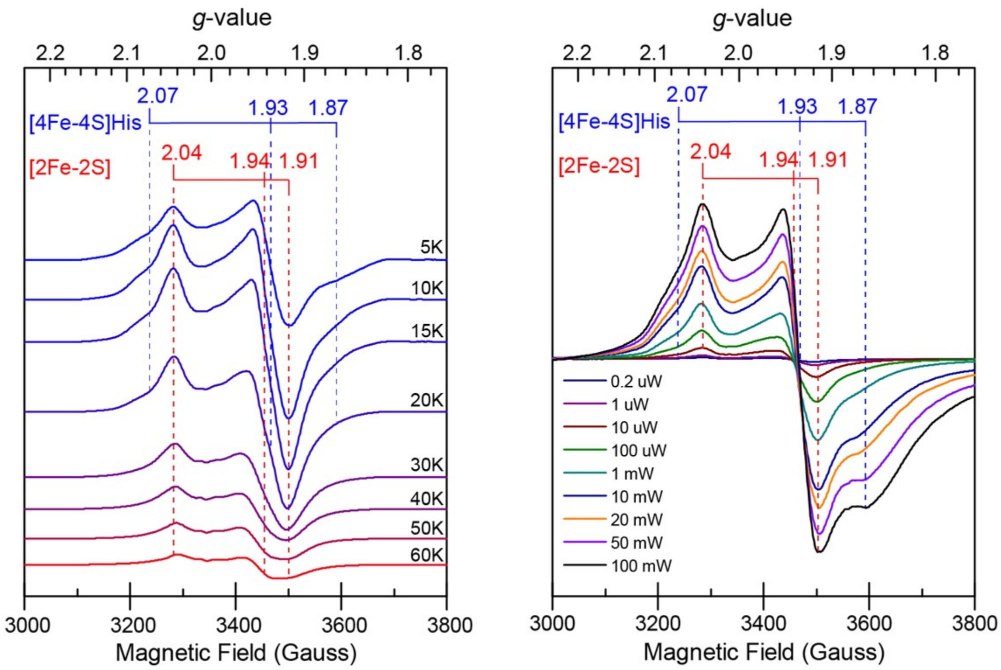

Figure S7. Temperature (left) and power (right) profiles of the *S* = ½ [4Fe-4S]His and [2Fe-2S] signals recorded for reduced DDHis. The *g*-value assignments of the individual clusters were obtained by spectral simulation and the [2Fe-2S] cluster signal could be isolated at temperatures above 30K due to temperature broadening of the [4Fe-4S]His cluster signal. The sample was prepared in 150 mM HEPES buffer, pH 8.8, 200 mM NaCl, 5% glycerol, and reduced with 33 mM dithionite. Measurement conditions: microwave frequency, 9.38 GHz; modulation frequency, 100 kHz; modulation amplitude, 10 Gauss. The temperature series was collected at a microwave power of 1 mW. The power series was collected at a temperature of 10K.

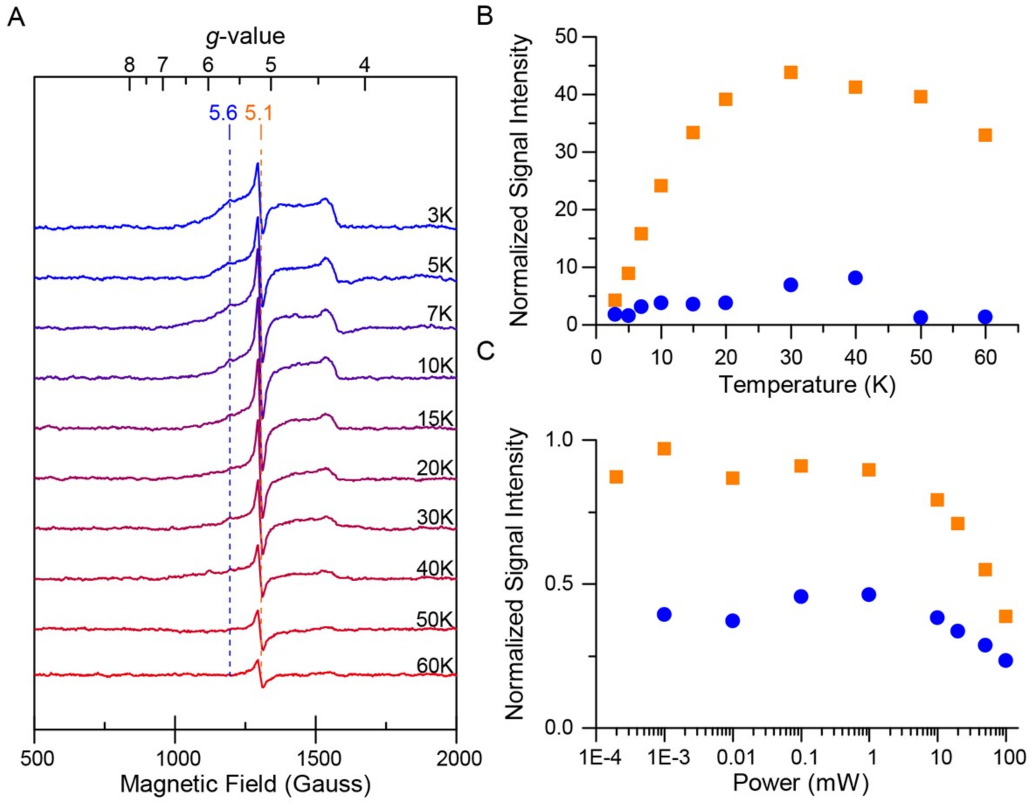

Figure S8. (A) Temperature profile of the low-field spectra recorded for reduced DDHis. (B) Temperature dependance of the g = 5.6 (blue circles) and g = 5.1 (orange square) signals. Signal intensities were measured by the signal height either referenced to the baseline (g = 5.6) or by the peak-to peak signal height (g = 5.1) and normalized by multiplying it by the collection temperature. (C) Power dependance of the g = 5.6 and g = 5.1 signals (normalized signal intensity = log (signal intensity/(microwave power)$^{0.5}$)). The sample was prepared in 150 mM HEPES buffer, pH 8.8, 200 mM NaCl, 5% glycerol, and reduced with 33 mM dithionite. Measurement conditions: microwave frequency, 9.38 GHz; modulation frequency, 100 kHz; modulation amplitude, 10 Gauss. The temperature series was collected at a microwave power of 1 mW. The power series was collected at a temperature of 10K.

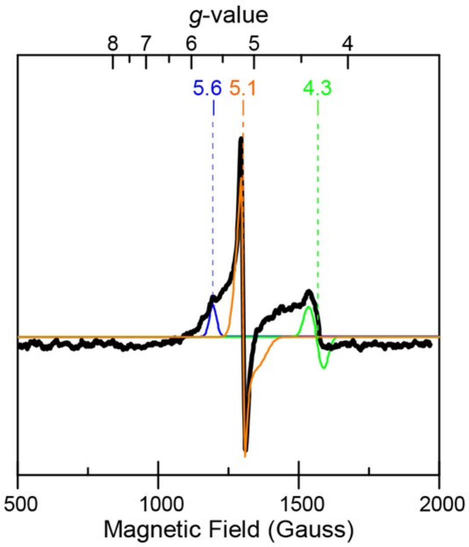

Figure S9. Simulation of the low-field, half-integer Kramer systems signals observed for reduced DDHis. Experimental spectrum (black trace), *g* = 5.6 simulation (blue trace, *S* = 3/2, ±3/2 doublet, E/D = 0.28), *g* = 5.1 simulation (orange trace, *S* = 7/2, ±3/2 doublet, E/D = 0.126). An additional feature at *g* = 4.3 that is attributed to an Fe^{3+} impurity, was also simulated (green trace, *S* = 5/2, ±3/2 doublet, E/D = 0.32).

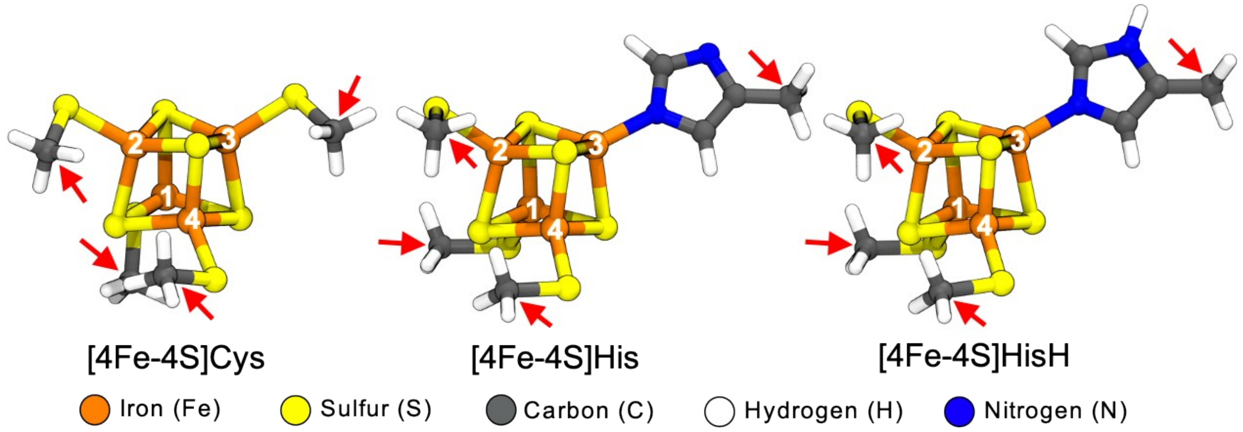

Figure S10. Model systems considered for BS-DFT calculations in this study. The models were created from the structure of the differentially ligated [4Fe-4S] cluster from the CpI crystal structure PDB ID: 3c8y. The [4Fe-4S]Cys cluster was created by mutating the imidazole ring with a methyl thiolate group. In the absence of the rest of the protein to tether the ligands, the C_b atoms were frozen (red arrows) to ensure that the ligand geometries do not assume dihedral angles that might be distant from the crystal structure. The Fe atoms are numbered 1-4 to keep track of spin pairing combinations with the site differentiated Fe atom being Fe3.

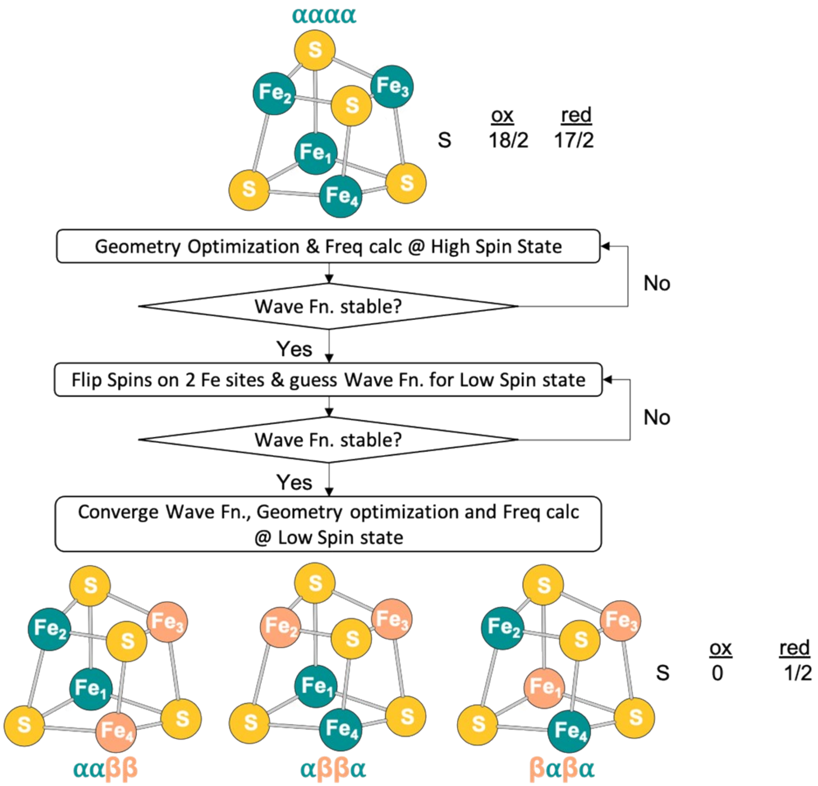

Figure S11. Schematic depicting the BS-DFT calculations. Calculations are initiated with a geometry optimization and frequency calculation at the high-spin state of the cluster with all Fe atoms having the same spin. This is followed by a calculation to ensure wave-function stability. Upon ensuring that the wave-function is stable, the spins on two of the Fe atoms are flipped to achieve the low-spin broken symmetry state, wherein there is ferromagnetic coupling within a pair of Fe atoms, which are in turn anti-ferromagnetically coupled. This spin-flip is achieved by providing suitable guesses for the spin and multiplicities for the Fe atoms and can result in three possible spin-pairing combinations as depicted above. A geometry optimization, frequency calculation and population analysis were performed at the low-spin broken symmetry state of the cluster.

Table S2. Calculated redox potentials of [4Fe-4S]HisH, [4Fe-4S]His and [4Fe-4S]Cys for three spin pairing combinations. Reduction potential values are versus theoretical absolute SHE at pH = 0.

Spin Pairing	[4Fe-4S]Cys ($E°$ in mV)	[4Fe-4S]His ($E°$ in mV)	[4Fe-4S]HisH ($E°$ in mV)	$E°$ of [4Fe-4S]His relative to [4Fe-4S]Cys ($\Delta E°$ in mV)[2]	$E°$ of [4Fe-4S]HisH relative to [4Fe-4S]Cys ($\Delta E°$ in mV)[2]
aabb	-1701.41	-1478.17	-1314.95	-223.25	-386.47
abba	-1533.86	-1535.01	-1269.66	1.15	-264.20
baba	-1631.20	-1519.27	-1306.74	-111.93	-324.46
Average[1]	-1622.16	-1510.82	-1297.12	-111.34	-325.04

[1]Averages over the three spin pairing combinations and differences of redox potentials for the [4Fe-4S]HisH and [4Fe-4S]His clusters compared to [4Fe-4S]Cys cluster are also listed.

[2]$\Delta E°$ [4Fe-4S]His = $E°_{\text{[4Fe-4S]Cys}}$ - $E°_{\text{[4Fe-4S]His}}$, and $\Delta E°$ [4Fe-4S]HisH = $E°_{\text{[4Fe-4S]Cys}}$ - $E°_{\text{[4Fe-4S]HisH}}$.

REFERENCES

1. P. W. King, M. C. Posewitz, M. L. Ghirardi and M. Seibert, *J. Bacteriol.*, 2006, **188**, 2163-2172.
2. W. W. Fish, in *Meth. Enzymol.*, Academic Press, 1988, vol. 158, pp. 357-364.
3. J. Riemer, H. H. Hoepken, H. Czerwinska, S. R. Robinson and R. Dringen, *Anal. Biochem.*, 2004, **331**, 370-375.
4. J. H. Artz, O. A. Zadvornyy, D. W. Mulder, S. M. Keable, A. E. Cohen, M. W. Ratzloff, S. G. Williams, B. Ginovska, N. Kumar and J. Song, *J. Am. Chem. Soc.*, 2019, **142**, 1227-1235.
5. S. Stoll and A. Schweiger, *J. Mag. Reson.*, 2006, **178**, 42-55.
6. H. Mundoor, T. Lee, D. G. Gann, P. J. Ackerman, B. Senyuk, J. van de Lagemaat and I. I. Smalyukh, *J. Appl. Phys.*, 2014, **116**, 063511.
7. S. F. Altschul, T. L. Madden, A. A. Schäffer, J. Zhang, Z. Zhang, W. Miller and D. J. Lipman, *Nucleic Acids Res.*, 1997, **25**, 3389-3402.
8. M. J. Frisch, G. W. Trucks, H. B. Schlegel, G. E. Scuseria, M. A. Robb, J. R. Cheeseman, G. Scalmani, V. Barone, G. A. Petersson, H. Nakatsuji, X. Li, M. Caricato, A. V. Marenich, J. Bloino, B. G. Janesko, R. Gomperts, B. Mennucci, H. P. Hratchian, J. V. Ortiz, A. F. Izmaylov, J. L. Sonnenberg, Williams, F. Ding, F. Lipparini, F. Egidi, J. Goings, B. Peng, A. Petrone, T. Henderson, D. Ranasinghe, V. G. Zakrzewski, J. Gao, N. Rega, G. Zheng, W. Liang, M. Hada, M. Ehara, K. Toyota, R. Fukuda, J. Hasegawa, M. Ishida, T. Nakajima, Y. Honda, O. Kitao, H. Nakai, T. Vreven, K. Throssell, J. A. Montgomery Jr., J. E. Peralta, F. Ogliaro, M. J. Bearpark, J. J. Heyd, E. N. Brothers, K. N. Kudin, V. N. Staroverov, T. A. Keith, R. Kobayashi, J. Normand, K. Raghavachari, A. P. Rendell, J. C. Burant, S. S. Iyengar, J. Tomasi, M. Cossi, J. M. Millam, M. Klene, C. Adamo, R. Cammi, J. W. Ochterski, R. L. Martin, K. Morokuma, O. Farkas, J. B. Foresman and D. J. Fox, *Journal*, 2016.
9. A. D. Becke, *Phys. Rev. A*, 1988, **38**, 3098.
10. S. Grimme, J. Antony, S. Ehrlich and H. Krieg, *J. Chem. Phys.*, 2010, **132**, 154104.
11. J. P. Perdew, *Phys. Rev. B*, 1986, **33**, 8822.
12. K. Eichkorn, O. Treutler, H. Öhm, M. Häser and R. Ahlrichs, *Chem. Phys. Lett.*, 1995, **240**, 283-290.
13. K. Eichkorn, F. Weigend, O. Treutler and R. Ahlrichs, *Theor. Chem. Acc.*, 1997, **97**, 119-124.
14. F. Weigend, *Phys. Chem. Chem. Phys.*, 2006, **8**, 1057-1065.
15. F. Weigend and R. Ahlrichs, *Phys. Chem. Chem. Phys.*, 2005, **7**, 3297-3305.
16. V. Barone and M. Cossi, *J. Phys. Chem. A*, 1998, **102**, 1995-2001.
17. M. Cossi, N. Rega, G. Scalmani and V. Barone, *J. Comput. Chem.*, 2003, **24**, 669-681.
18. L. Li, C. Li, Z. Zhang and E. Alexov, *J. Chem. Theory Comput.*, 2013, **9**, 2126-2136.
19. F. L. Hirshfeld, *Theor. Chim. Acta*, 1977, **44**, 129-138.
20. J. P. Ritchie, *J. Am. Chem. Soc.*, 1985, **107**, 1829-1837.
21. J. P. Ritchie and S. M. Bachrach, *J. Comput. Chem.*, 1987, **8**, 499-509.
22. W. Humphrey, A. Dalke and K. Schulten, *J. Mol. Graphics*, 1996, **14**, 33-38.
23. A. Kubas and P. Maszota, *Eur. J. Inorg. Chem.*, 2018, 2419-2428.
